# Diagnostics of allergic rhinitis under dupilumab therapy

**DOI:** 10.1007/s00405-024-08700-2

**Published:** 2024-05-09

**Authors:** Patrick Huber, Moritz Gröger, Clemens Stihl, Hanna Frankenberger, Mattis Bertlich, Frank Haubner, Donata Gellrich

**Affiliations:** 1 Department of Oto-Rhino-Laryngology, Head and Neck Surgery, Ludwig Maximilian University of Munich, Marchioninistraße 15, 81377 Munich, Germany; 2 Department of Dermatology and Allergology, Ludwig Maximilian University of Munich, Munich, Germany

**Keywords:** Allergy diagnostics, Biologics, Dupilumab, Allergic rhinitis, Chronic rhinosinusitis

## Abstract

**Background:**

Allergic rhinits is a prevalent condition, affecting a substantial proportion of the population. This study investigates the impact of ongoing biologic therapy, specifically with Dupilumab, on allergy diagnostics in patients with allergic rhinits.

**Methods:**

Various tests, including the Skin Prick Test, serum IgE levels and Allergy Screening Panels, were examined for their effectiveness in detecting sensitizations during biologic treatment.

**Results:**

The results indicate a significant decline in total IgE levels following biologic therapy initiation, aligning with previous findings on Dupilumab's inhibitory effects on IL-4 and IL-13. However, the specific IgE to total IgE ratio for major allergens was not significantly reduced. Comparing diagnostic tools, the Skin Prick Test demonstrates an impressive retention rate of sensitizations (98%) during Dupilumab treatment, outperforming the Allergy Screening Panel, which shows a 75% detection rate. Notably, the panel displays limitations in capturing lower sensitization levels.

**Conclusion:**

In summary, this study underscores that, despite the influence of biologic therapy on certain markers, standard allergy tests remain viable while emphasizing the importance of considering specific IgE levels rather than relying solely on CAP classes. The Skin Prick Test in particular proves to be a reliable tool for identifying sensitizations during Dupilumab treatment. The results offer valuable guidance for the diagnostic management of Allergic rhinits in individuals subjected to Dupilumab treatment.

## Introduction

Allergic rhinitis (AR) is a common pathological condition, affecting 20 to 30% of adults and up to 40% of children both in the United States and Europe [1]. Clinically, the disease is characterized clinically on exposure to allergen by one or more of the following symptoms: nasal itching, sneezing, nasal obstruction or congestion, rhinorrhea (anterior or posterior), and sometimes, reduction of sense of smell (hyposmia). The symptoms occur within minutes and can last for 1–2 h before improvement. Late-phase nasal symptoms can include nasal obstruction, hyposmia, postnasal mucous discharge, and nasal hyper-reactivity [2]. In 50–70% of cases AR is accompanied by conjunctivitis presenting with intense eye itching, hyperemia, watering, and occasionally, periorbital oedema [3].

AR in particular and allergic disorders are initiated by an allergic immune response to incorporated, especially inhaled allergens [2]. People with a genetic predisposition are at risk to become sensitized to harmless allergens through the activation of dendritic cells and T lymphocytes located in the nasal mucosa [4]. These cells act as antigen-presenting cells through binding the allergen to specific major histocompatibility complex (MHC) class II molecules. This MHC class II complex is then recognized by Th0 receptor and other costimulatory molecules, resulting in differentiation into Th2 CD4 + lymphocytes that produce cytokines like IL-4, IL-5, and IL-13 driving an IgE-mediated inflammatory immune response. The activation of antigen-specific Th2 lymphocytes also stimulates B-cell receptors, causing B-cell differentiation to antibody-producing plasma cells. Specific cytokines like IL-4 induce antibody class switching to IgE in B lymphocytes. The antigen-specific IgE binds to high-affinity IgE receptors on mast cells and basophils. On re-exposure of the allergen, the antigen is recognized by the IgE receptor. This results in the rapid release of preformed mediators such as histamine, leukotrienes and prostaglandins causing smooth muscle contraction, increased vascular permeability, and mucus secretion. In addition, enzymes such as tryptase are released causing tissue damage through the activation of matrix metalloproteinases. Also, the release of epithelial cell cytokines such as IL-25 and IL-33 further enhance the TH2 immune response [2, 5].

Taking into account the patients’ clinical history and symptom manifestation, to confirm a diagnosis of AR, specific IgE (sIgE) reactivity to airborne allergens must be proven. This is usually done either by skin-prick testing or by measurement of circulating allergen-sIgE antibodies usually via Carrier-Polymer-System-Test (CAP-Test). In addition molecular allergy diagnosis (MD) or component-resolved diagnosis (CRD) is used to differentiate between genuine sensitization to an allergenic source and sensitization resulting from cross-reactivity [6].

Observed closely, there are certain similarities between the pathomechanism of AR and Chronic Rhinosinusitis (CRS), especially of the Type 2 Endotype. CRS presents with two or more symptoms of nasal blockage, nasal discharge (anterior / posterior nasal drip), facial pain/pressure and/or hyposmia for ≥ 12 weeks [7]. While the exact details of the pathomechanism are yet not fully understood, the consensus is that CRS is a complex mucosal barrier disorder. Barrier penetration of pathogens results in a chronic inflammatory response typically utilizing type 1, 2 or 3 pathways alone or in combination. It has been demonstrated that patients with a predominant Type 2 inflammation present clinically as the phenotype of CRS with nasal polyps (CRSwNP) and tend to show higher resistance to current therapies, while having a higher probability of showing signs of other atopic diseases such as asthma. Type 2 inflammation is characterized by elevation in cytokines levels of IL-4, IL-5 and IL-13. In addition, elevated levels of innate lymphoid cells (ILC2), macrophages, and mast cells have also been detected [8]. These deeper understandings led to the development of biologic agents targeting specific cytokines or mediators of type 2 inflammation.

As of now, three biologics are currently approved in Germany as an add-on therapy for uncontrolled CRSwNP with minor differences in their respective areas of indication. The first and most frequently prescribed biologic that was approved for chronic sinusitis was Dupilumab (Dupixent®) in late 2019 [9]. It binds to the alpha subunit of the IL-4 receptor, which is not only part of the heterodimeric IL-4 receptor, but also of the IL-13 receptor, hence IL-4 and IL-13 signaling pathways are both inhibited, thereby attenuating type 2 inflammation [10].

Similarities in the underlying mechanisms of AR and Chronic Rhinosinusitis of the Type-2 Endotype suggest that biologics, known for their effectiveness in Type-2 immune responses, may also alleviate allergic symptoms in patients undergoing such treatment*.* Further, to our knowledge, little to none is known about the influence of Dupilumab on allergy diagnostics performed in the usual clinical setting. Therefore, the aim of this study was to assess the feasibility of detecting sensitization to inhaled allergens in individuals undergoing Dupilumab therapy. To achieve this goal, we compared comprehensive allergy diagnostics in patients with AR and CRSwNP prior to the start of Dupilumab treatment and under ongoing Dupilumab treatment. Our investigation aimed to determine whether the previously identified sensitizations persisted or became unddectable during the course of ongoing biologic treatment.

## Material and methods

Patient data was collected from the database of the Department of Oto-Rhino-Laryngology, Head and Neck Surgery of the Ludwig Maximilian University of Munich. The study was approved by the local ethics committee and the local data protection commissioner under the project number 22–0802. All patients provided written informed consent for the use of their parameters for scientific research and gave consent to publish these results.

The database was scanned for all patients with a current treatment with Dupilumab for CRSwNP, who also underwent an allergy diagnostic prior to starting treatment. Allergy diagnostics consisted of at least one of either Skin Prick Test (SPT), EuroImmun Multipanel or Carrier-Polymer-System-Test.

### Serum IgE

Total IgE-(IgE) levels were measured in 25 patients’ serum samples via immunonephelometry and were given in U/ml with a reference range of < 100 U/ml. Testing was performed by the central laboratory department of our clinic. Mean observational period for this collective was 28 months ranging from 5 to 42 months.

### Fluorescence enzyme immunoassay/carrier-polymer-system test

The fluorescence enzyme immunoassay (FEIA) method (UniCAP-FEIA; Thermo Fisher Scientific, Freiburg, Germany) was employed to detect sIgE reactivity with a commercially available test kit (Thermo Fisher Scientific, Freiburg, Germany). More specific sIgE to purified recombinants for rBet v 1 (birch), Phl p 1/p 5b (timothy grass), rOle e 1 (ash), nAmb a 1 (ambrosia), rPar j 2 (glasswort), nArt v 1 (mugwort), rPla l 1 (plantain), rDer p1, rDer p 2, rDer p 23 (house dust mite), rAlt a 1 (alternaria), rAsp f 1 (aspergillus) as well as native extracts for cat (e1), dog (e5) and cladosporium (m3) was analyzed. The results are given as CAP class (0: < 0.35 kUA/l; 1: ≥ 0.35–0.70 kU/L; 2: 0.71–3.50 kU/L;3: 3.51–17.50 kU/L; 4: 17.51–50.00 kU/L; 5: 50.01–100.00 kU/L; and 6: > 100.00 kU/L), although we considered a cut-off value for detection of sensitization for all tested reagents as ≥ 0.10 kU/L as suggested by the manufacturer. All sera were tested at the allergy laboratory of the ENT Department of the University of Munich using standardized methods in accordance with the manufacturer’s instructions. Mean observational period for this collective of 25 partients was 28 months ranging from 5 to 42 months.

### Allergy screening panel

As an Allergy Screening Panel EUROLINE (EUROIMMUN Medizinische Labordiagnostik AG, Lübeck, Germany) a solid phase, line-blot–type enzyme-linked immunoassay (colorimetric) consisting of 15 inhalation allergens with Dermatophagoides pteronyssinus, Dermatophagoides farinae, Dermatophagoides microceras, grass pollen mixture, birch, hazel, alder, ash, mugwort, plantain, cat, dog, horse, alternaria, and aspergillus was used. The results are presented semi-quantitatively in CAP classes (1: ≥ 0.35–0.70 kU/L; 2: 0.71–3.50 kU/L;3: 3.51–17.50 kU/L; 4: 17.51–50.00 kU/L; 5: 50.01–100.00 kU/L; and 6: > 100.00 kU/L). Testing was performed by the central laboratory department of our clinic. Mean observational period for this collective of 23 patients was 28 months ranging from 3 to 46 months.

### Skin prick test

A standardized SPT with Dermatophagoides pteronyssinus, Dermatophagoides farinae, grass mix, birch, hazel, alder, ash, mugwort, plantain, ragweed, cat, dog, alternaria, cladosporium, aspergillus, and positive and negative control (ALK-Abelló, Wedel, Germany) was documented in 20 patients before starting treatment with Dupilumab. The same test was performed during ongoing treatment after a mean treatment period of 31 months (ranging from 20 to 41 months) in the same patient group. The SPT was considered positive with a wheal ≥ 3 mm in diameter (I =  ≥ 3–4, II =  ≥ 4–5, III =  ≥ 5–6, and IV =  ≥ 6) in combination with histamine dihydrochloride solution at 1 mg/ml as positive control and allergen-free saline solution as negative control. It was read 20 min after application. The procedure and classification were in line with European standards and published guidelines. To account for any false positive or false negative results, all via SPT recorded sensitizations were cross-referenced with Carrier-Polymer-System-Test and therefore only sensitizations confirmed by means of serological diagnostics were considered as such.

### Statistical analysis

All statistical analyses were carried out using SigmaPlotTM 14.5 software tools (Systat Software, San Jose, USA). Differences were considered significant at *p* < 0.05.

## Results

43 Patients were identified who, in addition to being diagnosed with CRSwNP, showed symptoms of AR and therefore underwent some kind of allergy diagnostics prior to the treatment with Dupilumab. In these 43 patients, the same panel of allergy diagnostic tests were then repeated under the ongoing Dupliumab treatment. In 25 patients, an extensive serological processing was performed via CAP. In 20 of these patients, there was also a recorded SPT prior to start of treatment. In addition, 23 patients were identified who underwent an EuroImmun Multipanel allergy screening. Also, in 25 patients there was a regular registration of concentration of total serum IgE.

### Serum IgE

**Fig. 1:**
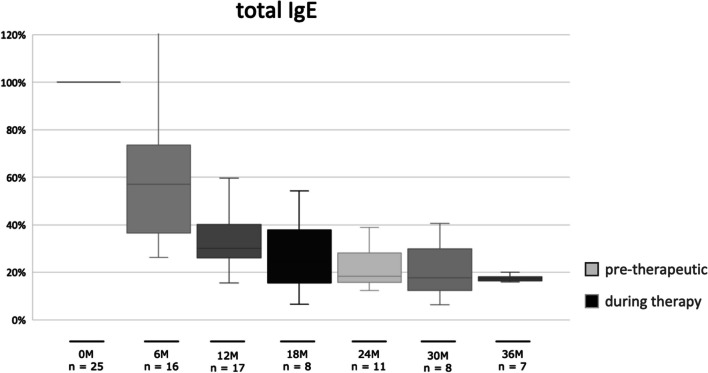
Mean value with standard deviation of tIgE during ongoing biologic treatment given in percentage of baseline value in 6th-month intervals with a mean observation period of 28 months

**Fig. 2:**
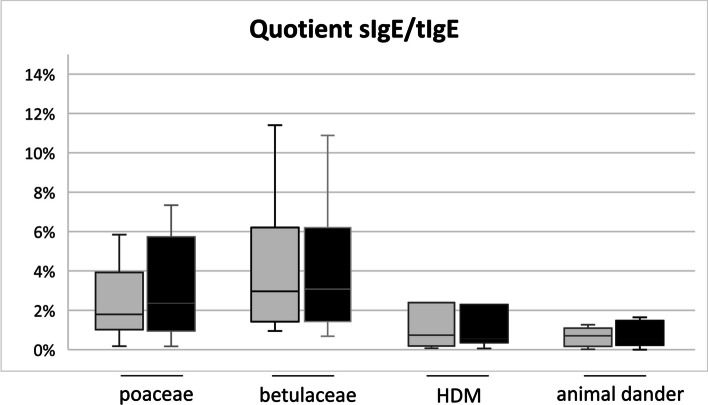
Mean value of the quotient of sIgE and tIgE given in percentage with standard deviation before [7] and during ongoing biologic treatment (black) for the allergens, poaceae (Phl p 1), betulaceae (Bet v 1), HDM (consisting of Der p 1, Der p 2 and Der p 23), and animal dander (dog native and cat native). Total number of 64 sensitizations with 18 sensitizations for poaceae, 14 sensitizations for betulaceae, 15 sensitizations for HDM and 18 sensitizations for animal dander

In 25 patients, a systematic assessment of total immunoglobulin E (tIgE) levels was conducted over an average observation period of 28 months, involving recurrent measurements taken at intervals ranging from 3 to 12 months. The investigation primarily centered focusedon quantifying the reduction in tIgE concentrations, expressed as a percentage relative to the initial baseline value. As shown in Fig. 1, the results indicate a substantial decline in tIgE levels, nearly approaching 50% reduction six months following the initiation of treatment. Subsequently, a plateau was observed at approximately 20% of the baseline tIgE levels after 24 months of continuous monitoring. Similar to total IgE, the levels sIgE against tested aeroallergens, expressed in kU/L, also dropped significantly: From 7.1 ± 7.6 kU/L to 2.2 ± 3.0 kU/L for poaceae (Phl p 1), from 9.1 ± 12.1 kU/L to 2.5 ± 3.8 kU/L for betulaceae (Bet v 1), from 12.5 ± 27.7 kU/L to 2.0 ± 4.3 kU/L for HDM (consisting of Der p 1, Der p 2 and Der p 23) and from 2.0 ± 3.3 kU/L to 0.4 ± 0.4 kU/L for animal dander (dog native and cat native). Comparing the quotient of sIgE of the respective antigen and tIgE before and during treatment with Dupilumab as depicted in Fig. 2 for the allergens HDM, betulaceae, poaceae and animal dander, no significant reduction was found respectively over a mean observation period of 28 months.

### Fluorescence enzyme immunoassay/carrier-polymer-system test

Within a cohort of 25 patients, sIgE reactivity against primary major allergens was assessed by the ImmunoCAP system before the commencement of therapeutic interventions and was later re-assessed during the Dupilumab treatment. Our focus was directed towards the principal allergen groups, which encompassed poaceae (grasses), betulaceae (birch trees), house dust mites, and animal dander. Among the patients, we identified 17, 14, 12, and 13 individuals who exhibited pre-existing sensitization, as indicated by a CAP class of ≥ 1, to poaceae, betulaceae, house dust mite, and animal dander, respectively.

**Fig. 3:**
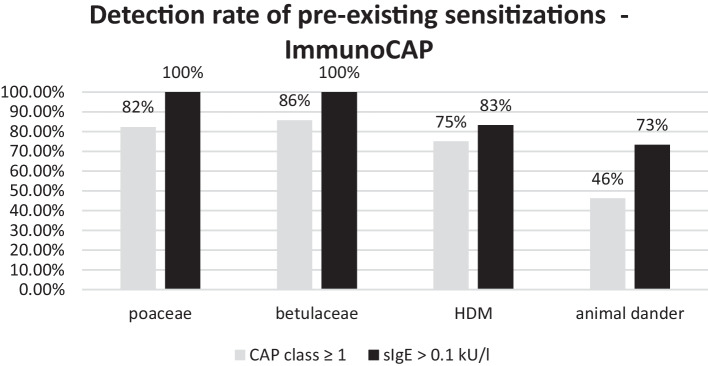
Detection rate of pre-existing sensitizations for poaceae (n = 17 with CAP class ≥ 1 and n = 17 with > 0.1 kU/l), betulaceae (n = 14 with CAP class ≥ 1 and n = 14 with > 0.1 kU/l), animal epithelia (n = 13 with CAP class ≥ 1 and n = 18 with > 0.1 kU/l) and HDM (n = 12 with CAP class ≥ 1 and n = 15 with > 0.1 kU/l) via serological sIgE measurement during biologic treatment with regard to CAP class ≥ 1 [7] and total value > 0.1 kU/l (black) given in percent

As delineated in Fig. 3, related to CAP class a relatively high rate of detection was observed for grass and birch pollen sensitization, standing at 82% and 86%, respectively. However, in the case of house dust mite sensitization, the CAP class system detected a pre-existing sensitization diagnosis in only 75% of the cases, and for animal dander, this rate was further reduced to 46%. As a CAP class rating of 0 does not equate to the absence of sIgE antibodies; rather, it suggests that the sIgE antibody levels are below the threshold for the lowest CAP class. However, when employing a detection limit of > 0.1 kUA/l as a cut-off value, our observations reveal that evidence of pre-existing sensitization to the primary allergens in grasses and birch trees remains detectable in 100% of cases under dupilumab therapy. Furthermore, in the case of house dust mites, such sensitization was present in 83% of the cases, and sensitization to animal dander was still discernible in 73% of cases during the course of Dupilumab therapy. Overall, the recognition rate of a CAP class rating of ≥ 1 was 73% (41 of 56 sensitizations), while the recognition rate of > 0.1 kUA/l was 89% (57 of 64 sensitizations).

### Allergy screening panel

**Fig. 4:**
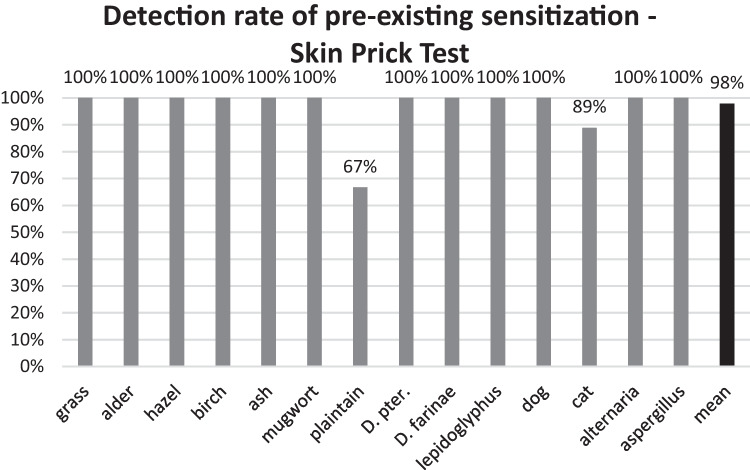
Detection rate of pre-exisiting sensitizations for grass mix (n = 18), birch (n = 13), hazel (n = 13), alder (n = 14), ash (n = 0, data not shown), mugwort (n = 3), plantain (n = 6), HDM (n = 27 consisting of D. pteronyssinus, D. farina and D. microceras), animal dander (n = 12 consisting of cat, dog and horse), aspergillus (n = 1), alternaria (n = 3) via Allergy Screening Panel during biologic treatment, given in percent

Data from an Allergy Screening Panel consisting of 15 aeroallergens were available in 23 patients. A total of 123 sensitizations were documented prior to initiation of treatment with Dupilumab. 90 (73%) of them could be retrieved after a mean treatment period of 27 months. 22 of 33 sensitizations which could not be retrieved, were classified as CAP class of 1 prior to start of treatment with a biologic. Figure 4 displays the recognition rate by the Allergy Screening Panel for sensitization against HDM, grass, birch and animal dander (72%, 80%, 92%, and 67% respectively).

### Skin prick-test

**Fig. 5:**
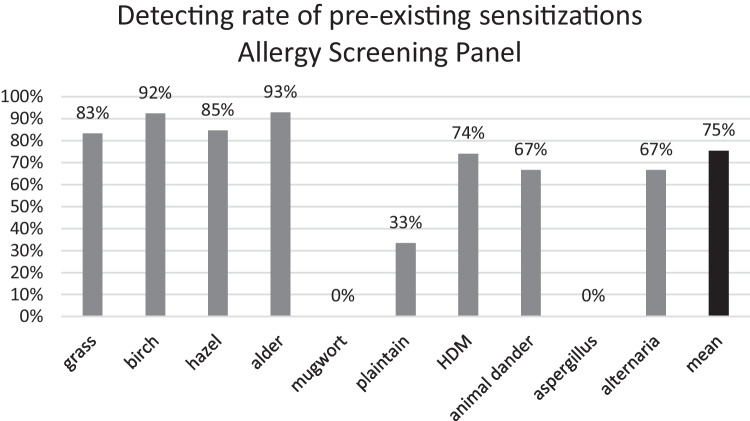
Detection rate of pre-existing sensitizations for grass mix (n = 16), alder (n = 12), hazel (n = 12), birch (n = 12), ash (n = 3), mugwort (n = 1), plantain (n = 3), dermatophagoides pteronyssinus (n = 7), dermatophagoides farinae (n = 7), lepidoglyphus (n = 2), cat (n = 9), dog (n = 7), alternaria (n = 4), aspergillus (n = 1) via Skin Prick Test during biologic treatment, given in percent

In 20 patients with an ongoing biologics-treatment, SPT was performed prior to start of Dupilumab therapy. In this cohort a total of 96 sensitizations were identified by SPT. The SPT with identical test allergens was repeated after a mean period of 31 months (ranging from 20 to 41 months) of therapy with Dupilumab. As shown in Fig. 5, 94 (98%) of the total 96 previously found sensitizations, could be recorded by SPT under ongoing treatment with Dupilumab. Only one sensitization to plantain in one patient and one sensitization to cat in another patient was not detectable by SPT under Dupilumab.

## Discussion

One of the main pitfalls in allergy diagnostics is the influence of medication taken prior to testing. To prevent confounding effects on test outcomes, the intake of systemic antihistamines, for instance, is recommended to be discontinued for a minimum of seven days preceding in vitro or in vivo testing [11]. However, there are no recommendations nor empirical data concerning the management of monoclonal antibodies within this context. Nevertheless, this issue is on the rise as the utilization of Dupilumab and other biologics expands in the management of CRSwNP [12, 13]. Given the frequent co-occurrence of AR alongside CRSwNP of type 2 endotype [14] there is a discernible increase in the prevalence of conducting allergy diagnostics within the framework of Dupilumab treatment.

Via its subcutaneous route of administration Dupilumab achieves peak serum concentration within 3 to 7 days. Notably, it exhibits a relatively long half-life, approximately ranging from 17 to 20 days [15]. With a recommended administration rate of once every 14 days, a waiting period prior to allergy testing does not seem feasible only leaving the possibility of performing allergy diagnostics under ongoing biologic therapy. The present data aimed to assess potential limitations of allergy diagnostics if performed under Dupilumab treatment which is in our clinic the therapeutic choice for nearly 95% of the patient population with uncontrolled CRSwNP.

A comprehensive analysis of immunological parameters was available in a cohort of 25 patients, assessed over an average observation period of 28 months. Evaluation of tIgE levels revealed a substantial decline in IgE concentrations, approaching nearly 50% reduction six months following the commencement of biologic therapy. This is in line with published data where Dupilumab through its inhibiting of IL-4 and IL-13 has been shown to lead to a substantial reduction in tIgE typically occuring within the first few months of treatment [16]. Further, our data confirm that Dupilumab does not only reduce tIgE, but also sIgE against airborne allergens as already shown with respect to several food allergens by Spekhorst et al. [17]. Interestingly, evaluation of the quotient of sIgE and tIgE concerning various major inhalant allergens demonstrated no statistically significant reduction of the quotient of sIgE in tIgE in all analyzed allergens e.g. HDM, betulaceae, poaceae, and animal dander. The pivotal question is whether reducing tIgE concentrations might lead to a decrease in sIgE antibodies to the point where it becomes challenging to detect systemic sensitization using the ImmunoCAP test for allergy diagnostics. The concern is that this situation could result in overlooking important sensitizations with clinical significance.

Therefore, were further investigated the data of sIgE sensitizations. Considering recovery rates, as indicated by the presence of a CAP class ≥ 1 for IgE antibodies directed against the primary and substantial allergens encompassing poaceae, betulaceae, HDM, and animal dander a relatively high detection rate of 82% and 86% is evident for grass and birch pollen allergens respectively. In stark contrast, the CAP class system detected pre-existing sensitization to house dust mite (HSM) in only 75% of the cases and to animal dander in a mere 46% of cases. This implies that, conversely, a house dust mite sensitization might go unnoticed in 25% of cases, and a sensitization to animal dander could be overlooked in 54% of cases. It is imperative to recognize that a CAP class of 0 does not inherently signify the absence of sIgE antibodies. Indeed, when employing a detection threshold of > 0.1 kUA/l as the cut-off value, evidence of pre-existing sensitization to the major allergens in grasses and birch trees remains detectable in 100% of cases for each allergen under the influence of Dupilumab. Furthermore, in the context of house dust mite sensitization, this remains true in 83% of the cases, and sensitization to animal dander remains discernible in 73% of cases during the course of Dupilumab therapy. Overall, with regard to CAP class ≥ 1 a recognition rate of 73% was demonstrated, with stark contrast to a recognition rate of 89% when employing a detection threshold of > 0.1 kUA/l. This implies that when conducting allergy diagnostics via measurement of sIgE in the presence of Dupilumab, special emphasis should be placed on the absolute measurement of sIgE levels expressed in kUA/l. Relying solely on the categorization into CAP classes does not provide a robust basis for making conclusive assessments.

Upon establishing the viability of detecting sensitizations under treatment with biologics to a range of allergens through the quantification of sIgE directed against various allergenic components via the ImmunoCAP test, the question at hand pertains to the comparability and effectiveness of this method when compared to the conventional practices of skin prick testing and the utilization of standard Allergy Screening Panels, which are typically employed in initial allergy assessments.

Preceding the onset of biologic therapy, a total of 110 sensitizations to 15 aeroallergens were recorded employing the Allergy Screening Panel. These sensitizations were expressed semi-quantitatively in terms of CAP classes equal to or greater than 1. Following an average treatment duration of 28 months, it was observed that 83 of these sensitizations (75%) remained detectable, while more than a fourth of preexisting sensitizations were no longer recognizable. It is imperative to note that this diagnostic approach solely provides an assessment of sensitization categorized in CAP classes without offering quantification in terms of sIgE levels. Notably, among the 27 sensitizations that were not recognized, 16 initially fell into a CAP class of 1 prior to biologic treatment, implying a potential limitation of the screening tool, particularly in cases of lower sensitization levels. In contrast, the data derived from the Skin Prick-Test (SPT) revealed that 94 out of the initial 96 sensitizations (98%) endured throughout the patients' course of biologic therapy. Only two sensitizations, deemed of minimal clinical significance and related to plantain and cat allergens, were not detectable in two distinct patients. It is crucial to underscore that our investigation specifically assessed sensitizations rather than allergies. Therefore, one might hypothesize, that the recognition rate of clinically manifest allergies might be even higher, as most undetected sensitizations had a pre-therapeutic low sensitization level making them more likely to lack clinical relevance [6].

Our findings suggest that SPT may offer a more consistent and comprehensive assessment of sensitizations under biologic treatment, as confirmed by its superior recognition rate of sensitizations in comparison to the Allergy Screening Panel. While the applicability of these observations to other commercially available test kits remains speculative, it is conspicuous that SPT stands out as a precise diagnostic tool in the context of detecting sensitizations to allergens during Dupilumab treatment.

Another limitation of this study is the uneven temporal follow-up of the patient sample. While a minimum follow-up of 20 months for the ImmunoCAP test seems sufficient, the occasionally shorter follow-up timepoints for SPT and Allergy Screening may introduce a bias and skew the results.

While Dupilumab has demonstrated its efficacy as an adjunct treatment for patients with uncontrolled persistent asthma and comorbid AR [18], it is essential to emphasize that the primary objective of this study was not to assess the clinical benefits or therapeutic effects of Dupilumab in the context of AR. While this topic certainly warrants further investigation, the primary focus of our study was to evaluate whether ongoing treatment with Dupilumab has any impact on allergy diagnostics conducted in routine clinical practice. Therefore, we can assert that, while acknowledging the inherent limitations and challenges, allergy diagnostics during antibody therapy with Dupilumab certainly remains viable and feasible.

## Data Availability

The data that support the findings of this study are available from Patrick Huber upon reasonable request.
